# Inline Inspection with an Industrial Robot (IIIR) for Mass-Customization Production Line †

**DOI:** 10.3390/s20113008

**Published:** 2020-05-26

**Authors:** Zai-Gen Wu, Chao-Yi Lin, Hao-Wei Chang, Po Ting Lin

**Affiliations:** 1 Department of Mechanical Engineering, National Taiwan University of Science and Technology, Taipei 10607, Taiwan; geass0931927669@gmail.com (Z.-G.W.); joecool1024@gmail.com (C.-Y.L.); chw1994@gmail.com (H.-W.C.); 2 Center for Cyber-Physical System Innovation, National Taiwan University of Science and Technology, Taipei 10607, Taiwan

**Keywords:** 3D scanner, 6R robot arm, automatic optical inspection, coordinate transformations

## Abstract

Robots are essential for the rapid development of Industry 4.0. In order to truly achieve autonomous robot control in customizable production lines, robots need to be accurate enough and capable of recognizing the geometry and orientation of an arbitrarily shaped object. This paper presents a method of inline inspection with an industrial robot (IIIR) for mass-customization production lines. A 3D scanner was used to capture the geometry and orientation of the object to be inspected. As the object entered the working range of the robot, the end effector moved along with the object and the camera installed at the end effector performed the requested optical inspections. The detailed information about the developed methodology was introduced in this paper. The experiments showed there was a relative movement between the moving object and the following camera and the speed was around 0.34 mm per second (worst case was around 0.94 mm per second). For a camera of 60 frames per second, the relative moving speed between the object and the camera was around 6 micron (around 16 micron for the worst case), which was stable enough for most industrial production inspections.

## 1. Introduction

With the rapidly developing trend of Industry 4.0, industrial robots have been widely used in various applications, such as automotive manufacturing lines [1], semiconductor production lines [2], etc. For the goal of autonomous control of industrial robots, various methodologies have been developed to achieve high-accuracy robot manipulation. It has been expected that an Industry 4.0 production line is massively customized. In a mass-customization production line, there exist various kinds of products with different geometrical shapes. There may not be a general solution for automation. Thus, sensing and manipulation with a high degree of freedom became essential for handling these mass-customized products.

In a traditional production line, additional workers or devices may be needed to align or position their products in the conveying system. Inline inspection, therefore, is required to be able to recognize the position and orientation of an inspected object without the need for additional human workers and positioning devices. Moreover, the conveying system usually needs to stop for a couple of seconds to perform stable optical inspections. Stoppage of the conveying system decreases the overall production yield. It is desirable to develop an inline system that is capable of performing inspection of a product in a moving conveyor.

Recently, researchers have developed several robotics systems for picking or inspecting target objects in a moving conveyor or a production line. Han, et al. [3] determined the optimal path planning of a robot arm to pick randomly placed objects in a moving conveyor in terms of minimizing the pick-and-place time in the reachable range of the robot arm. They found that the effectiveness of object picking is related to how the objects are distributed in the conveyor. Anwar, et al. [4] developed some visual servo methodologies to move a camera at the end effector of a robot arm to face the inspection surface on an object. The end effector position was determined based on an image-based Jacobian approach. Abbood, et al. [5] used a camera and some image-processing techniques to identify the shape of an object in a moving conveyor. A low-cost system based on a simple robot arm with plastic gears and servo motors was built to demonstrate the concept of picking objects at a slow moving speed of less than 9 cm/s.

One of the most important features in the inline inspection system for mass-customization production lines is high-accuracy manipulation. Juan and his colleagues [6,7] measured the absolute position errors of the end effector of a 6-revolute-joint (6R) robot arm in the working space by laser. Accordingly, the error field of the end effector in the working space was parametrically modeled by kriging and was utilized to compute the corrected coordinates of the end effector. With the high accuracy of the laser measurement (i.e., ~0.001 mm), the absolute accuracy of the end effector was improved from ~1 mm to ~0.05 mm. However, the experimental setup of laser measurement was very time consuming.

To make the 3D position measurement of the end effector more efficient, Juan and his colleagues [8,9] measured the distances from the end effector to three different cable encoders. These three length measurements were then used to compute the 3D position of the end effector by trilateration. With a lower accuracy of the cable encoder (i.e., ~0.1 mm), the accuracy of the absolute positioning of the end effector reached the range of ~0.5 mm. Lin, et al. [10] also showed how multilateration could be used to estimate the end effector position when more than 3 cable encoders were used to measure their distances to the end effector. Shih and Lin [11] started to investigate how the end effector positioning under various levels of payloads could be improved using trilateration based on three cable encoder measurements and coordinate correction based on kriging models of error fields.

Cable encoder measurements show good potential on 3D positioning of industrial robots but they are not suitable for in situ measurements. Shih, et al. [12] developed a vision-based correction of end effector positioning in planar motions. A 2D camera was used to compute the transformation between the camera coordinates and the end effector coordinates in a given plane. Any target position of the end effector in the plane could then be computed with its coordinate in the live image. This approach was good for in situ measurement and robot manipulation but it only worked for positioning in a given plane and not suitable for moving target positions.

Wu, et al. [13] utilized a 3D scanner to capture the geometry and orientation of an object, which was randomly placed in a moving conveyer. Since the moving speed and direction of the conveyer was known, a 6R robot was able to move along with the moving object as it passed through the working range of the robot. The developed stereoscopic object tracking could be utilized for the application of inline inspection of randomly placed objects in a moving conveyer with known speed and direction. Wu, et al. [14] presented the development and implementation of inline inspection based on stereoscopic object scanning and object tracking by a 6R robot.

In this paper, a method of inline inspection with an industrial robot (IIIR) and its applications in mass-customization production lines are presented. Section 2 introduces the experimental setup and the required methodologies for matching the coordinates of the industrial robot and the 3D scanner. Section 3 explains how the camera position could be estimated based on the computer-aided design (CAD) model of a given object and the 3D point cloud of the object captured under the 3D scanner. Section 4 shows the experimental process and the results. Finally, the conclusion is given in Section 5.

## 2. Experimental Setup and Initialization of the Inline Inspection with a 6R Robot

**Figure 1:**
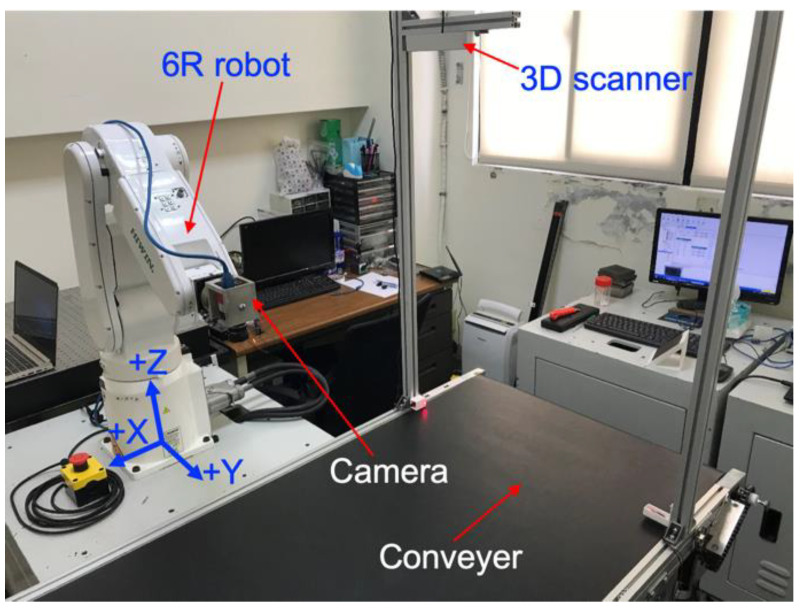
Experimental setup of the inline inspection (X-Y-Z is the origin of the robot coordinate).

Figure 1 shows the experimental setup of the developed inline inspection system [13]. A 3D scanner (i.e., stereovision camera with two pixel sensors in a resolution of 2 micron) was installed at the top of the entrance of a conveyer. The moving speed of the conveyer was denoted as V and was given as 33.8 mm/s. The moving direction of the conveyer was along the +X direction of robot coordinate, which could be seen in Figure 1. As an arbitrarily shaped object entered the conveyer, its 3D geometry and orientation would be captured by the 3D scanner. The 3D point cloud acquired by the 3D scanner would be used to calculate the desired positions and orientations of the end effector of the industrial robot. Accordingly, the end effector was desired to move along with the object and perform optical inspections without stopping the conveyer. This paper will present the required methodologies to estimate the end effector coordinates for the proposed inline inspection.

**Figure 2:**
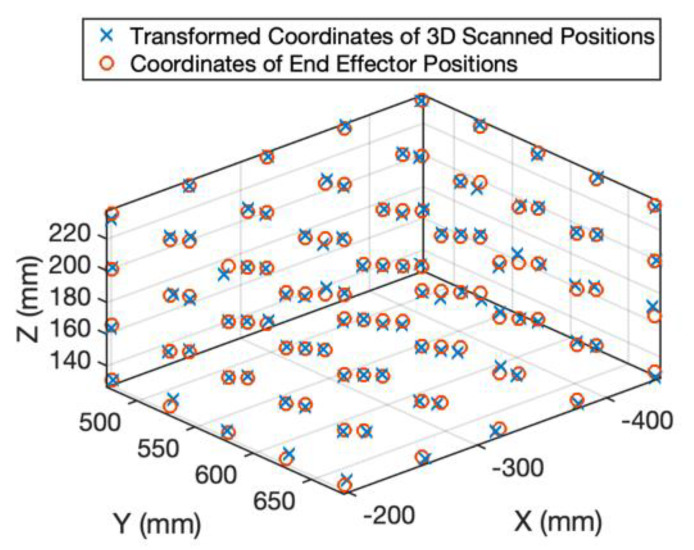
The coordinates of the end effector positions and the transformed coordinates of the 3D scanned positions.

To match the 3D scanner coordinate, S, and the robot coordinate, R, the transformation from S to R, denoted as TSR, should be computed. The end effector of the robot was moved to N different positions within the inspection range of the 3D scanner. In this paper, N=100 and N points were uniformly distributed, as shown as the red circles in Figure 2. The $i^{th}$ end effector position observed in R was denoted as PiR. Accordingly, the 3D scanner was used to captured these end effector positions and $i^{th}$ end effector position captured in S was denoted as QiS. The $i^{th}$ transformed 3D scanned position was denoted as QiR and should satisfy the following equation:

(1) QiR=TSR⋅QiS

Since the end effector coordinate PiR should be closed to the transformed 3D scanned coordinate QiR, the transformation could then be estimated by least square approximation (LSA).

The Equation (1) can be rewritten as the following matrix form:

(2) [Qi,1RQi,2RQi,3R1]=[TS,11RTS,12RTS,13RTS,14RTS,21RTS,22RTS,23RTS,24RTS,31RTS,32RTS,33RTS,34R0001]⋅[Qi,1SQi,2SQi,3S1]

where each vector was formulated in the homogeneous coordinate. To determine the unknown parameters, TS,pqR for p=1,2,3 and q=1,2,3,4, the data from all N experiments was put together producing the following equation:

(3) [Q1,pRQ2,pR⋮QN,pR]=[Q1,1SQ1,2SQ1,3S1Q2,1SQ2,2SQ2,3S1⋮⋮⋮⋮QN,1SQN,2SQN,3S1]⋅[TS,p1RTS,p2RTS,p3RTS,p4R]

for p=1,2,3. Equation (3) is rewritten as the following:

(4) $$\omega_{p}=\Omega \cdot \tau_{p}$$

where $\omega_{p}$ is a N×1 vector that contains the $p^{th}$ coordinate of every QiR; Ω is a N×4 matrix that contains all the coordinates of every QiS; $\tau_{p}$ is a 4 × 1 vector that contains the components of the $p^{th}$ vrow of TSR. Based on LSA, the unknown parameters in $\tau_{p}$ could be estimated by:

(5) $$\tau_{p}={\left(\Omega^{T}\cdot \Omega \right)}^{-1}\cdot \Omega^{T}\cdot \omega_{p}$$

**Figure 3:**
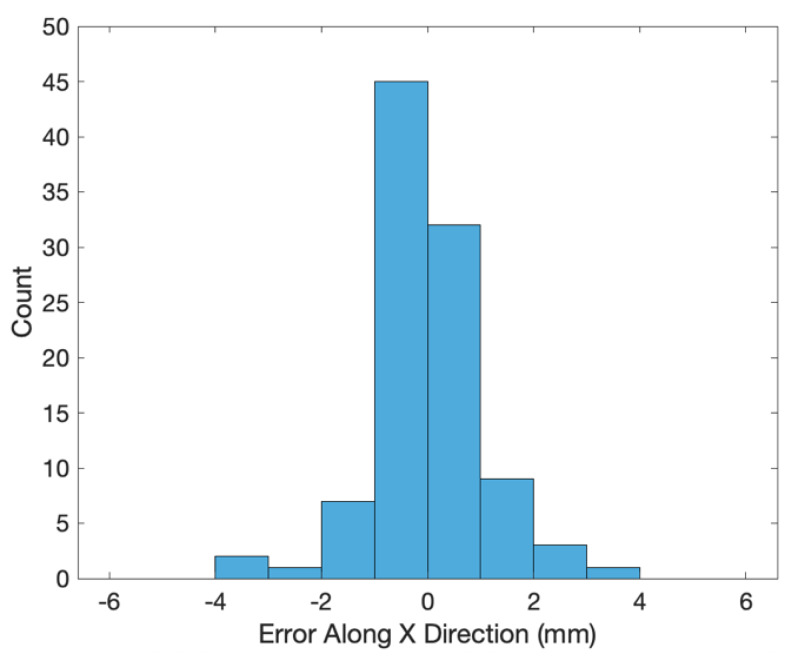
Histogram of errors along X direction between the end effector coordinates and transformed coordinates of the 3D scanned positions.

**Figure 4:**
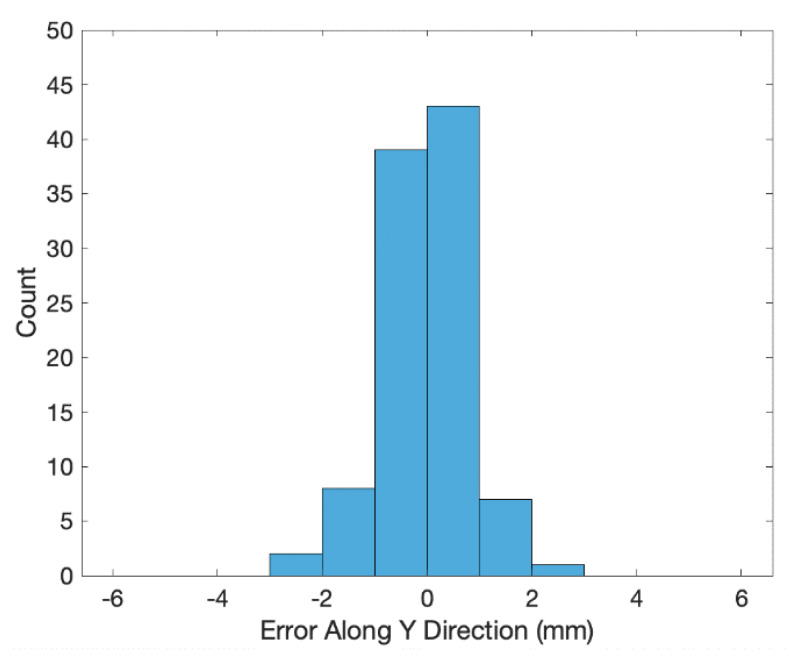
Histogram of errors along Y direction between the end effector coordinates and transformed coordinates of the 3D scanned positions.

**Figure 5:**
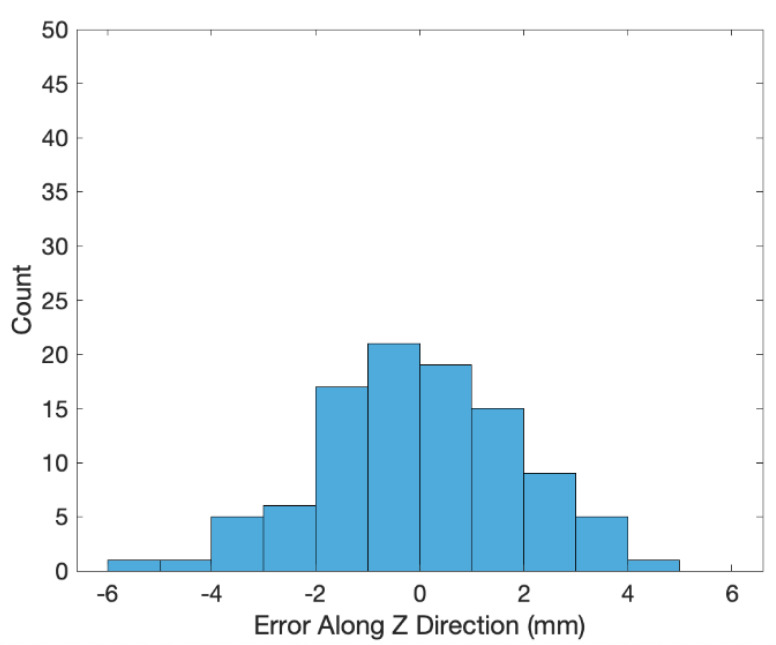
Histogram of errors along Z direction between the end effector coordinates and transformed coordinates of the 3D scanned positions.

**Figure 6:**
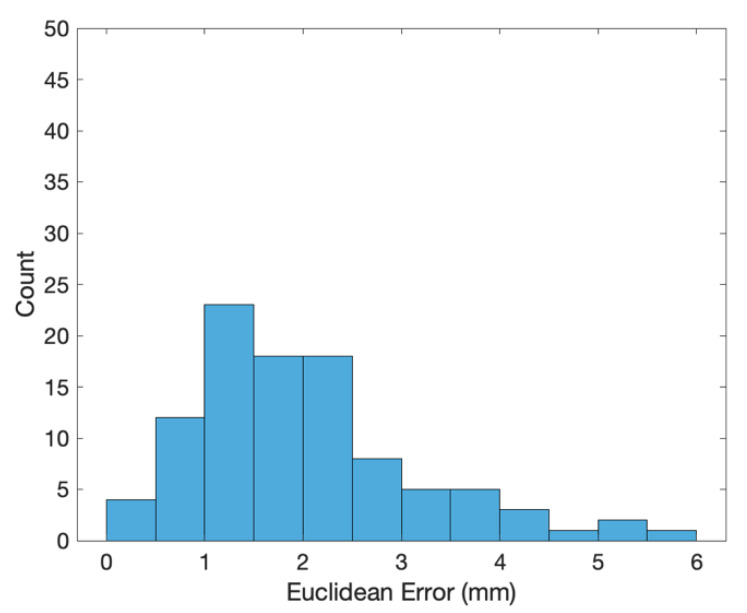
Histogram of Euclidean errors between the end effector coordinates and transformed coordinates of the 3D scanned positions.

The transformed coordinates of the 100 end effector positions measured by the 3D scanner are shown as the blue crosses in Figure 2. The errors (i.e., coordinates of the end effector positions minus the transformed ones of the 3D scanned positions) along the X, Y and Z directions are shown in Figure 3, Figure 4 and Figure 5, respectively. The averages and standard deviations of the errors along the X, Y and Z directions are listed in Table 1. Since the 3D scanner is less accurate in the Z direction than the X and Y directions, the standard deviation of errors along the Z direction is greater than those along the X and Y directions. Figure 6 shows the Euclidean errors between the end effector positions and transformed ones of the 3D scanned positions. Based on the averaged value of Euclidean errors listed in Table 1, the error between PiR and QiR was around 2 mm, which was mostly caused by the lower accuracy of the 3D scanner along the Z direction.

## 3. Estimation of the End Effector Coordinates for the Inline Inspection

**Figure 7:**
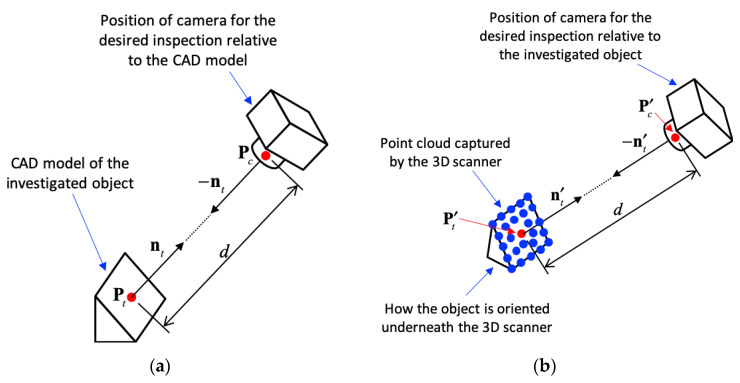
Position of the camera for the desired inspection: (**a**) relative to the CAD model of the investigated object; (**b**) relative to the point cloud of the object captured by 3D scanner.

Figure 7 illustrates the position of the camera for the desired inspection. Suppose the CAD model of the investigated object was known, as shown in Figure 7a. The camera was expected to take a picture facing a target point, $P_{t}$, on the object along the direction of $-n_{t}$, where $n_{t}$ stands for the normal vector of object surface near $P_{t}$. The CAD model and the desired camera position were saved in the database as the cyber model for later object recognition and positioning of camera. The distance from $P_{t}$ to the center of the camera lens, $P_{c}$, was given as d. Therefore, $P_{c}$ could be determined by:

(6) $$P_{c}=P_{t}+d\cdot n_{t}$$

The transformation from $P_{t}$ to $P_{c}$ could be extended to more complicated conditions. This paper only focuses on the translation along the direction of $n_{t}$ with a distance of d.

As the true object entered the conveyer as shown in Figure 1, the geometry and orientation of the object was captured by the 3D scanner, as shown as the blue points in Figure 7b. Iterative closest point (ICP) [15] determined the transformation, $T_{ICP}$, from the known CAD model to the point cloud obtained from 3D scanner. ICP, which was first introduced by Besl and McKay [15] in 1992, iteratively finds the minimal distance between two sets of points, lines, or surfaces. Later on, Fischler and Bolles [16] used random sample consensus (RANSEC) to improve the accuracy of 3D data sampling. Wahl, et al. [17] was able to capture the geometrical features from the sampled 3D data and analyze the histogram of the point features. Rusu, et al. [18] then introduced a fast point feature histogram (FPFH) to speed up the process of point feature analysis. This paper uses the FPFH to estimate the transformation between the known CAD model and the captured 3D point cloud.

Based on the transformation of the model matching, the target point on the object, denoted as $P_{t}^{\prime }$, was then found near the point cloud captured from the 3D scanner and was given by:

(7) $$P_{t}^{\prime }=T_{ICP}\cdot P_{t}$$

The normal vector of the surface near $P_{t}^{\prime }$ was determined by LSA and was denoted as $n_{t}^{\prime }$. Therefore, the position of the camera to perform the desired inspection could be computed by:

(8) $$P_{c}^{\prime }=P_{t}^{\prime }+d\cdot n_{t}^{\prime }$$

Consider the object entered the conveyer at time of t=0, the coordinate of $P_{c}^{\prime }(t=0)$ may not be inside the working range of the robot. It’s necessary to estimate at what time the object would move into the working range of the robot so that the desired inspection could be performed. In our implementation, the conveyer moved along the +X direction of the robot coordinate. Therefore, the Y- and Z-components of $P_{c}^{\prime }$ did not change with time. Only the X-component of $P_{c}^{\prime }$, denoted as $P_{c,x}^{\prime }(t)$, varied with time t and could be written as:

(9) $$P_{c,x}^{\prime }(t)=P_{t,x}^{\prime }+d\cdot n_{t,x}^{\prime }+V\cdot t$$

where $P_{t,x}^{\prime }$ and $n_{t,x}^{\prime }$ stand for the X-components of $P_{t}^{\prime }$ and $n_{t}^{\prime }$, respectively. Suppose the $P_{c,x}^{\prime }(t=t_{1})$ entered the working range of the robot and $P_{c,x}^{\prime }(t=t_{2})$ moved out of the working range. The inspection should be done within the time interval of $\left[t_{1},\;t_{2}\right]$. From Equation (9), the limits of the inspection time could be determined by:

(10) $$t_{1}=\frac{P_{x,\min}-P_{t,x}^{\prime }-d\cdot n_{t,x}^{\prime }}{V}$$

(11) $$t_{2}=\frac{P_{x,\max}-P_{t,x}^{\prime }-d\cdot n_{t,x}^{\prime }}{V}$$

where $P_{x,\min}$ and $P_{x,\max}$ represent the minimal and maximal X-coordinates of the working range of the robot, respectively. Finally, the coordinates of the end effector for following the object and performing the desired inspection were determined, i.e., $P_{c}^{\prime }(t=t_{1})$, $P_{c}^{\prime }(t=t_{1}+\Delta t)$, …, $P_{c}^{\prime }(t=t_{2})$. At each time t, the desired position and orientation of the end effector were computed based on the inverse kinematics in the motor control system.

## 4. Experiment and Result of the Proposed Inline Inspection

**Figure 8:**
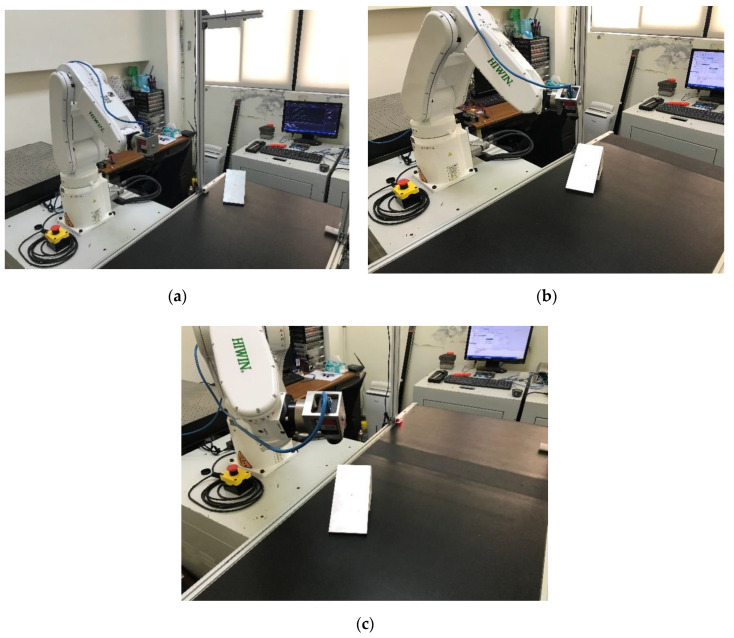
Demonstration of the experimental process: (**a**) An object was placed underneath the 3D scanner at a random position on the conveyer; (**b**) the industrial robot moved the camera to the desired position to face perpendicularly to the inspected surface on the object; (**c**) the camera moved with the movement of the object without stopping the conveyer.

The experimental process of the presented methodology is shown in Figure 8. An object to be inspected entered the conveyer at the beginning of the experiment, as shown in Figure 8a. The geometry and the orientation of the object were then captured by the 3D scanner. Suppose the CAD model of the object was known, the transformation between the captured point cloud and the CAD model in the database was estimated by ICP, as explained in Section 3. The target inspection point on the surface of the object, $P_{t}^{\prime }$, was then calculated as well as the normal vector of the inspected surface, $n_{t}^{\prime }$. Given the desired distance between the camera and the target point, *d*, the desired positions of the camera, $P_{c}^{\prime }$, could then be calculated.

Considering the known moving speed of the conveyer and the working range of the industrial robot, the camera could reach the desired positions between time $t_{1}$ and time $t_{2}$, as shown in Equations (10) and (11), respectively. Figure 8b shows the moment of time $t_{1}$ where the camera moved to the desired position of $P_{c}^{\prime }(t_{1})$. The industrial robot started to follow with the movement of the object to create near-zero relative movement between the camera and the moving object, as shown in Figure 8c. The inline spection was then performed before the object moved out of the reachable range at time $t_{2}$.

**Figure 9:**
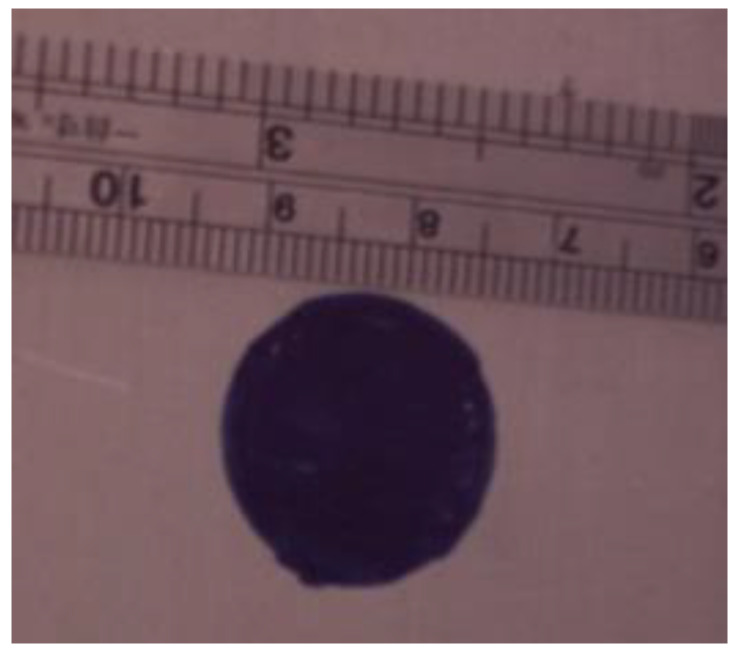
The circular mark on the flat plate.

**Figure 10:**
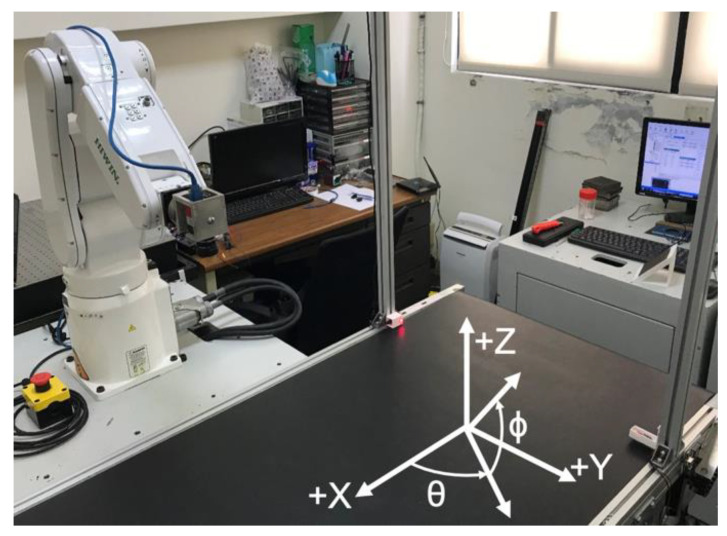
The directional angle θ and the tilting angle ϕ of the normal vector $n_{t}^{\prime }$ of the object.

In the experiments of the inline inspection, a flat plate with a circular mark, as shown in Figure 9, was to be inspected. The center of the circular mark was the target point for inspection. The normal vector of the plate had a directional angle, θ, from the X axis and a tilting angle, ϕ, from the X-Y plane, as shown in Figure 10. The area of the circular mark was 301.69 mm^2^. The area of the circular mark would be measured during the object following and inline inspection to show the accuracy of the optical inspection. The relative movement between the camera and the moving plate would be investigated to show the stability of the inline inspection system.

**Figure 11:**
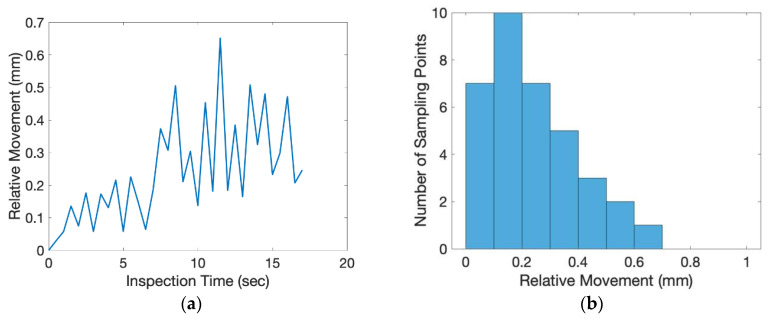
The relative movement of the center of the circular mark between each consecutive inspection image: (**a**) analysis with respect to time, (**b**) histogram of analysis.

**Figure 12:**
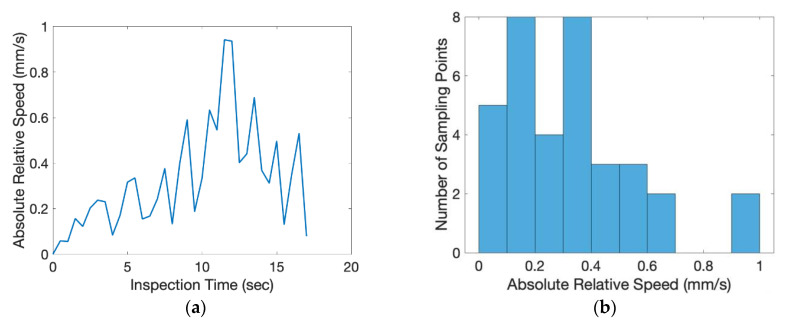
The absolute relative speed of the center of the circular mark between each consecutive inspection image: (**a**) analysis with respect to time, (**b**) histogram of analysis.

**Figure 13:**
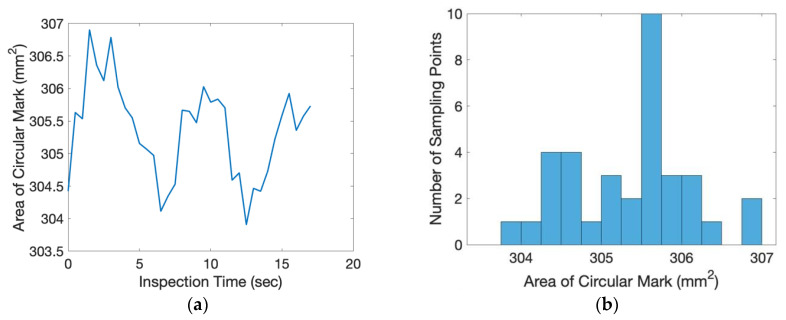
The measured area of the circular mark during the inspection process: (**a**) analysis with respect to time, (**b**) histogram of analysis.

**Figure 14:**
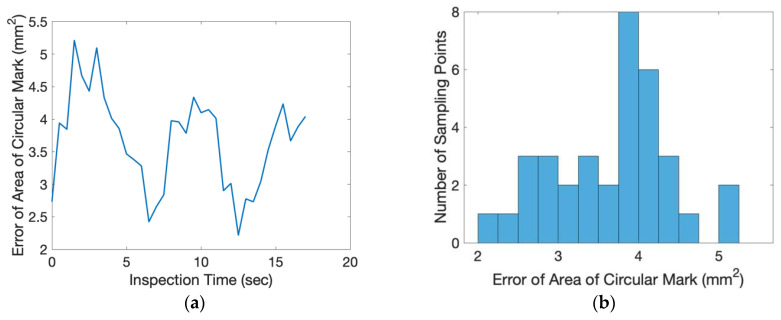
Error of the measured circular area during the inspection process: (**a**) analysis with respect to time, (**b**) histogram of analysis.

In this experiment, the directional angle θ of the inspection surface was −45° and the tilting angle ϕ was 28.7°. The exact position of the object was randomly placed on top of the conveyer. The scale factor of the inspected image was 0.1865 (mm/pixel). From the beginning to the end of the inspection process, one inspection image of the flat plate was taken for every 0.5 s. The relative movement of the center of the circular mark between each consecutive image was measured and shown in Figure 11. The average relative movement of center was 0.2460 mm and the maximum relative movement was 0.6521 mm. Taking derivative with time, the absolute relative speed of the center was shown in Figure 12. The average speed was 0.3358 mm/s and the maximum speed was 0.9420 mm/s. For a camera of 60 frames per second (fps), the relative moving speed between the object and the camera was around 6 micron (around 16 micron for the worst case), which was stable enough for most industrial production inspections. During the inspection process, the area of the circular mark was measured and shown in Figure 13. Compared to the true circular area, 301.69 mm^2^, the error of the area measurement during the inspection process is shown in Figure 14. The average error was 3.6695 mm^2^ (1.21% of error) around and the maximum error was 5.2122 mm^2^ (1.73% of error). The results of the presented experiment are listed in Table 2.

A comparison between the presented IIIR and some other existing methods that were developed in the past few years is given in Table 3. The investigated systems were developed for either pick-and-place applications [5,12,19,20] or production inspections (i.e., [4] and this work). For planar manipulations [5,12], image-based object recognition would be enough; on the other hand, 3D scanning was utilized for recognizing the objects that were either randomly placed (i.e., [20] and this work) or had arbitrary shapes [19]. The challenges of the robot manipulations increased as the objects were placed in a moving platform (i.e., [5] and this work). The comparison in Table 3 showed that the presented IIIR delivered a unique ability to recognize randomly placed objects in a moving platform with good accuracy (i.e., relative speed between camera and object = 0.3358 mm/s = 5.6 micron/frame for a 60-fps camera), which is important for the inline inspection in a mass-customization production line.

## 5. Conclusions

This paper has presented a method of inline inspection with an industrial robot (IIIR) that carries a camera to move along with moving object and performs the desired optical inspections in mass-customization production lines. The developed inline inspection system integrated multiple technologies, including a 3D scanning, embedded system, coordinate transformation, robot control and machine vision. In our implementation, the relative speed between the moving object and the camera and the speed was around 0.34 mm per second (worst case was around 0.94 mm per second). For a camera of 60 frames per second, the relative moving speed between the object and the camera was around 6 microns (around 16 microns for the worst case), which was stable enough for most industrial production inspections. Furthermore, the average error of the inspection measurement was round 1.21% (worst case was 1.73% of error). The developed system could be extended to various kind of inline operations for automation applications in Industry 4.0.

The main contribution of this paper was the integrated system of a 3D scanner for recognizing the position and orientation of a moving object, an industrial robot for moving to the desired position to perform inline inspection without stopping the conveyor, and a series of numerical methods for matching the coordinates of the 3D scanner and the robot arm, and calculating the desired end effector positions for inline inspections. The developed system was able to perform accurate object tracking without using costly measurement devices such as a high-speed camera or laser tracker. Therefore, it could be widely applied to various kinds of automation production lines but also highly applicable for small- and medium-sized enterprises.

## Figures and Tables

**Table 1:** Averages and standard deviations of the errors between the end effector coordinates and transformed coordinates of the 3D scanned positions.

Errors	Average (mm)	Standard Deviation (mm)
Errors along X direction	−0.0012	0.9844
Errors along Y direction	0.0002	0.8312
Errors along Z direction	−0.0002	1.9227
Euclidean errors	2.0041	1.1577

**Table 2:** Experimental results of the presented process of inline inspection with an industrial robot (IIIR).

Analyzed Data	Average	Standard Deviation	Worst Case	Unit
Relative movement	0.2460	0.1532	0.6521 (max. value)	mm
Absolute relative speed	0.3358	0.2261	0.9420 (max. value)	mm/s
Area of circular mark	305.3595	0.7186	306.9022 (measure with max. error of 5.2122 mm^2^)	mm^2^
Error of area of circular mark	3.6695	0.7186	5.2122 (max. value)	mm^2^

**Table 3:** Comparison between the proposed IIIR and other existing methods/technologies.

Methodologies/Technologies	Manipulation Types	Positioning Methods	Object Conditions	Accuracy/Performance
Automated sorting of a robotic vision system [5]	Pick and place using a 4 degrees of freedom (DOF) robot arm	Image-based shape recognition	Randomly placed in a moving conveyor with a known and constant speed (<9 cm/s)	92% success rate of shape sorting
Pick and place of deformable objects [19]	Pick and place with a 6R robot arm	3D scanning and parameter optimization	Randomly placed in a container	98% success rate of picking the pork loins
Vision-based end effector positioning [12]	Pick and place with a 6R robot arm	Vision-based planar positioning	Randomly placed in a planar surface	Positioning error = 0.42 mm
Planar object picking based on a deep learning network [20]	Pick and place with a 6R robot arm	3D scanning and coordinate matching (based on deep learning)	Randomly placed in a container	Positioning error = 3.6 mm
Quality inspection using depth-free image-based visual servo [4]	Target tracking with a 6R robot arm and performing inspections	Image-based visual servo	Placed in a fixed platform	Positioning error = 5.5 micron
Presented IIIR in this work	Object following with a 6R robot arm and performing inspections	3D scanning and coordinate matching	Randomly placed in a moving conveyor with a known and constant speed (33.8 mm/s)	Relative speed between a 60-fps camera and object = 5.6 micron/frame
